# Colorectal Cancer Screening: Impact of COVID-19 Pandemic and Possible Consequences

**DOI:** 10.3390/life11121297

**Published:** 2021-11-26

**Authors:** Isabelle Harber, Dania Zeidan, Muhammad N. Aslam

**Affiliations:** Department of Pathology, University of Michigan Medical School, Ann Arbor, MI 48109, USA; isharber@umich.edu (I.H.); zeidania@umich.edu (D.Z.)

**Keywords:** colorectal cancer, CRC screening, COVID-19, Spanish flu, pandemic

## Abstract

Colonoscopy procedure has been the key screening method to detect colorectal cancer (CRC). As a fatal disease, CRC needs early detection. The COVID-19 pandemic caused screening tests (colonoscopy) to be halted and delayed. As a result, there could be dire consequences such as later-stage or missed diagnosis or greater mortality. This report will analyze scientific literature pertaining to interrupted CRC screenings due to COVID-19 while drawing historical parallels from the 1918 flu pandemic. We conducted literature searches in the PubMed database as well as in Google Scholar. One of the main lessons learned from the 1918 flu pandemic was to employ social distancing to stop the spread of the virus. So, the global response at the start and peak of the COVID-19 pandemic was decreased hospital visits for any non-emergency cases. That led to a halt and delays in cancer (including CRC) screenings. The Medical community predicted this lag will cause more CRC cases and deaths in the future. However, reorganizing and changing screening method strategies were helpful during the ongoing pandemic. In conclusion, COVID-19 greatly affected CRC screening, including how we view the future of CRC screening. We can learn from this prospect to better prepare for future pandemics or other public health crises.

## 1. Introduction and Background

In 1918, the world faced a public health crisis known as the 1918 influenza pandemic or also known as the Spanish flu. This H1N1 influenza virus killed an estimated 50 million people worldwide [1,2,3]. Now, about 103 years later, the world is facing yet another devastating public health crisis—COVID-19, a respiratory disease caused by the novel coronavirus SARS-CoV-2 [4]. According to the John Hopkins University COVID-19 tracker, as of 25 November 2021, COVID-19 infection has been diagnosed in over 259 million people and has killed around 5.2 million people worldwide since the beginning of the pandemic. The United States of America (USA) alone is one of the worst-affected countries, with over 48 million cases and 775,628 deaths due to COVID-19 so far [5]. According to the CDC data, despite all of the scientific advancements, COVID-19 cases and casualties in the USA have surpassed the 1918 influenza pandemic death toll (of 675,000) [2]. We are still in the midst of the COVID-19 pandemic as COVID-19 infections in the United States, and several parts of the world (in hotspots zones) are rising even after the launch of successful COVID-19 vaccination programs. Table 1 breaks down the comparison of two pandemics, cases caused by 1918 Flu and COVID-19 and fatalities due to these infections.

The COVID-19 infectious disease poses a serious threat to people with underlying chronic conditions, including cancer. One of the most serious threats and concomitant manifestations of the surge in COVID-19 cases was a sharp decline in the screening of different types of cancers, i.e., breast, cervix, lung, and colon/rectum, at the early stages of the COVID-19 pandemic [6,7]. In addition to a decrease in the screening procedures, COVID-19 impacted colorectal cancer (CRC) care during the pandemic. This effect was due to the inadequacy of health care systems, lack of preparedness, availability of resources, and increased burden of COVID-19 cases [8]. The focus in this literature review will be on the effect of the COVID-19 pandemic on CRC screening.

CRC is the third most frequently diagnosed cancer type in the USA [9]. Risk factors of CRC include older age, obesity, alcohol use, tobacco use, family history of CRC, hereditary genetic mutations, and presence of inflammatory bowel disease (the duration, extent, age at onset, and the relation of primary sclerosing cholangitis of the IBD further affect CRC risk) [10]. CRC arises from the formation of polyps, unusually large growths in the colon or rectum. It is possible for polyps to progress to tumors, and expression profiles of proteins like cytokeratins and mucins can help determine prognosis and targeted therapy for specific types of colonic cancer [11]. There are many different types of screening tests that can detect these colonic polyps. These screening tests are important preventive measures for early CRC detection. Early detection of CRC (by way of screening tests) is of vital importance in preventing CRC and catching it before it progresses to a more severe stage. In fact, because regular screening has increased so much within the past 20–30 years, the death rate of CRC has been decreasing [9]. And of course, the decrease in death rate is also because of improved treatment options and targeted therapies [12]. Similarly, dietary and chemopreventive measures also play a role in decreasing the overall incidence of CRC [13,14].

Currently used screening methods include invasive tests such as colonoscopy and flexible sigmoidoscopy; or minimally invasive tests like colon capsule endoscopy (CCE); and noninvasive tests–the guaiac-based fecal occult blood test (FOBT), fecal immunochemical test (FIT), FIT-DNA test, and computed tomography colonography (CTC) [15]. Colonoscopy is arguably the most effective (gold-standard) screening method currently available since the invention of fiber-optic endoscopes in the late 1960s [16,17]. It gives physicians the ability to examine the entire colon and rectum of a patient and remove polyps at the same time [17]. The first rigid sigmoidoscope was available in 1805 (before the start of 1918 pandemic) to examine the last part of the colon for screening purposes [18]. Sigmoidoscope has also evolved and has been modernized [19,20]. Flexible sigmoidoscopy proceeds by the same mechanism as colonoscopy but does not examine the entire colon [19,20], while CCE is a relatively new method in which a pill with a camera takes pictures of the gastrointestinal tract when swallowed [21,22]. The noninvasive tests listed above are all stool tests except for CTC, testing patients’ stool samples to detect occult blood, or DNA [15]. Table 2 below further highlights the differences between the types of screening tests [15] and their pertinence during the COVID-19 pandemic.

At the beginning of the COVID-19 pandemic, there was a complete halt in CRC screenings due to efforts to minimize person-to-person contact and to conserve hospital and medical resources for those exposed to COVID-19 [6]. Unfortunately, the postponement of these screening tests for patients has had and will continue to have dire consequences, including more cases of CRC—potentially more severe ones—and additional CRC deaths that would have been prevented otherwise. With delayed or altogether missed screenings comes the potential for missed opportunities to detect polyps, diagnose CRC, and get more treatment for patients diagnosed.

There are no clear data in the existing literature showing the effects of the 1918 flu pandemic on the incidence or mortality of colorectal cancer during or after the pandemic. Myrskylä et al. published a study in 2013, explaining the early life exposure to the 1918 influenza pandemic and cause of death later in life (old age). The study showed that early age influenza exposure increased old-age mortality through noncancer causes (including respiratory and cardiovascular diseases) [3]. Furthermore, Wingo et al. published a paper in 2003, presenting long-term trends in cancer mortality in the USA from 1930 to 1998. It was found that the mortality rate of colon cancer declined over seven decades, going from 15.9% in the 1930s to 10.4% in the 1990s, with the highest mortality of 16.9% in the 1940s [23].

Siegel et al. documented long-term trends in CRC incidence from 1975–2010 and mortality rate from 1930–2010 in the USA. They concluded that CRC was the most common cause of cancer death in the USA in the late 1940s and early 1950s and after that, the mortality rate began to fall in the early 1950s [24]. In another publication, Siegel et al. presented that the age-specific risk of a CRC diagnosis fell for consecutive generations in the first half of the twentieth century but increased back to the level of those born in the late 1800s for current birth cohorts [25]. By reviewing this literature, it is difficult to make any connection to the after-effects of the 1918 flu pandemic on the incidence or mortality of CRC. What impact COVID-19 will have on the incidence and mortality of CRC is ultimately unclear, but we can predict from the suggestive tell-tale signs along with prediction models and population data.

There have been several papers published since the COVID-19 pandemic began about COVID-19-related CRC-screening delays, along with how these delays could impact future CRC incidence, mortality, and screening methods. Many of these papers are highly quantitative and some contain models relating to the prediction of future CRC statistics, including the number of CRC screening tests done pre-pandemic versus in the pandemic itself and after. This review will aim to highlight the important statistics and information regarding delayed CRC screenings, providing people in the public health field a holistic understanding of COVID-19′s impact on CRC screening, including those who may not necessarily have a wider knowledge of CRC. This review will also place the context of the pandemic’s impact on CRC concerning how CRC was affected by pandemics and epidemics of the past, such as the 1918 influenza pandemic. This comparison makes it more distinct from other literature reviews.

## 2. Materials and Methods

**Figure 1:**
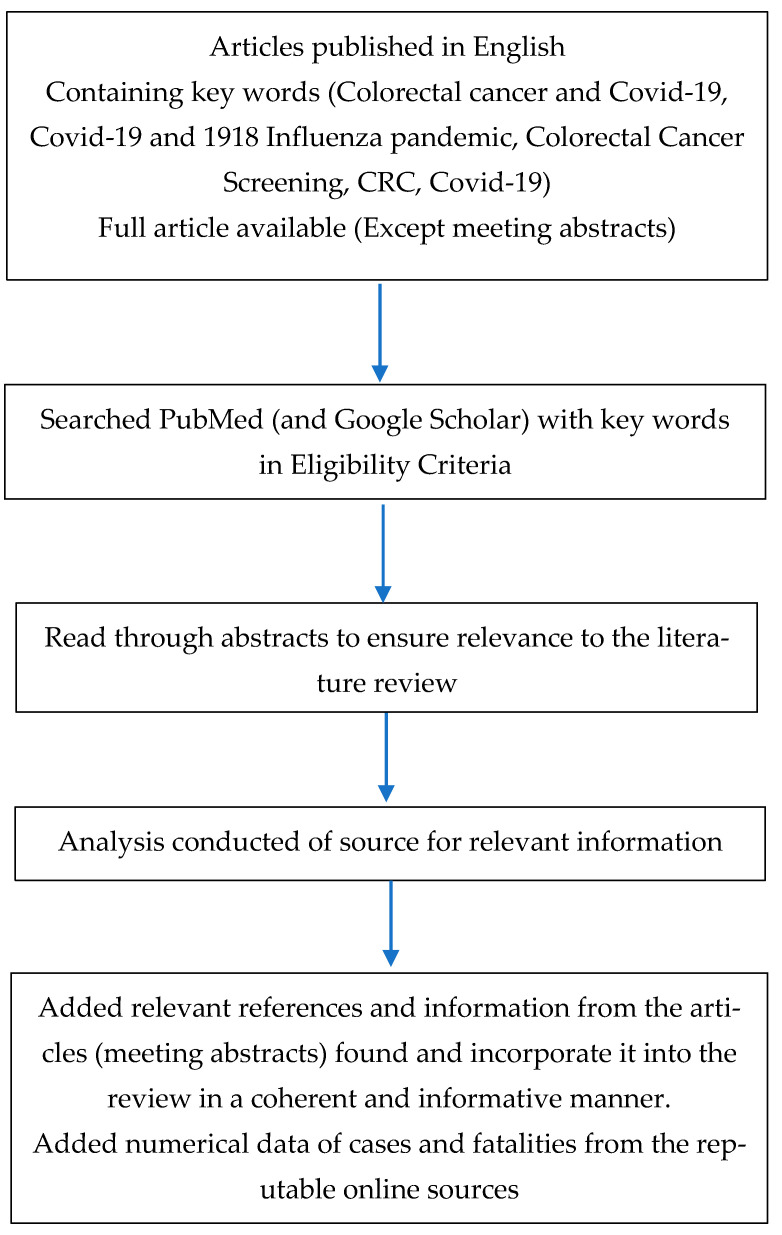
A flowchart describing eligibility criteria of relevant sources employed.

In order to conduct the literature review, we first described our topic and created an outline to better specify the types of sources and the content of information we would cite and reference in the literature review. We conducted searches in PubMed and Google Scholar databases using search terms including, but not limited to, “colorectal cancer COVID-19,” “colon cancer screening COVID-19,” “COVID-19 and 1918 influenza pandemic” and “colorectal cancer and Spanish flu.” We also used these and other search terms, as needed, in Google searches. With Google searches, we focused on finding additional information from websites of organizations, government agencies, or other reputable sources including meeting abstracts. Below is a flowchart summarizing the methods for retrieving and selecting scientific articles from the PubMed database (Figure 1).

## 3. Immediate Delays in CRC Screening and Their Predicted Consequences

In the early stages of COVID-19 pandemic, most healthcare systems around the world were not adequately prepared to handle what was about to come. CRC-screening tests were halted as national and regional lockdowns began, not to mention that healthcare centers needed to prioritize COVID-19 patients. Several studies have shown how much CRC-screenings decreased in the first few months of the pandemic. One study in the Netherlands compared the number of endoscopies conducted between 15 March 2020, and 25 June 2020, to the number of endoscopies conducted in the same period of 2019, using a database that gathers data from 20 hospitals in the Netherlands. It was found that there were only 9776 endoscopies performed in the 2020 period compared to 19,296 in the 2019 period. Also, colonoscopy procedures decreased by 55% (from 12,219 to 5609) at the same time [26]. Similarly, a retrospective analysis on surveillance colonoscopy (in patients at high-risk for CRC) in South Australia revealed that there was a 51.1% decrease in surveillance colonoscopy procedures from April–June 2019 compared to April–June 2020, the period where the region faced the most difficulty due to COVID-19 [27]. The USA experienced greater decreases in CRC screenings. In one study involving 20 medical centers, encompassing more than 28 million patients total, it was determined that there was a 38.4% decrease in March 2020 compared to March 2019 and an 84.5% decrease was observed in April 2020 compared to April 2019 [28]. Furthermore, another study (unpublished, presented at virtual UEG week 2021) of hospitals in Spain found that there was a 40.4% decrease in CRC diagnosis from the pre-pandemic period (of March 2019 to March 2020) to the pandemic period (of March 2020 to February 2021). On top of this, the author of the study said that there were fewer tumors diagnosed during the pandemic period by screening and more by the presenting symptomology [29]. It was also reported that there was trend toward a higher presentation of stage 4 tumors in the pandemic period. This increase in stage 4 tumors suggests that delays in the ability to get screened within the pandemic period led to later stages of cancer being diagnosed in patients—a highly worrisome change. Overall, in many different countries, similar declines in CRC screening were observed.

There are multiple modeling studies published that have predicted the consequences of delayed CRC screening and diagnosis, and most of these predictions are rather alarming. For example, in a population-based modeling study done in the United Kingdom (UK), in which data of 24,975 CRC patients were analyzed, it was estimated that there would be an additional death toll of 1445 to 1563 people up to five years post-diagnosis due to delays in screening and consequent late diagnoses [30]. Similarly, a microsimulation modeling study was done in Canada estimating the clinical long-term impact of these delays and interruptions in CRC screening. The model projected that in terms of primary screening, a six-month suspension could result in an increase in CRC incidence by 2200 cases along with an excess of 960 deaths [31]. In the British Journal of Cancer, it was estimated using microsimulation models that without catching up to missed screenings, the death rate for colorectal cancer will increase to 2.5 per 100,000 people in the next decade [32]. Furthermore, another modeling study predicted between the year 2020–2023, there would be about 1,176,942 to 2,014,164 less CRC screening due to delays in screening in the U.S [33]. It was also predicted that the reduction in cancer screening will lead to 8346 to 12,894 fewer CRC diagnoses and 6113 to 9301 fewer early-stage CRC diagnoses during the same period [33]. This portrays the fatal consequence of late diagnosis as early detection of CRC increases the overall chance of patient survival. All-in-all, these models outline a common narrative of a predicted increase in CRC incidence and mortality rates due to interruptions and delayed screenings of CRC. These screenings are crucial in cancer prevention as they allow for removals of precancerous lesions, preventing the advancement of the lesion to early stages of cancer [32,33]. Without these early screening and detection methods, the consequences could be fatal.

During the 1918 flu pandemic, CRC screenings, as we know them today, were not even around. For example, it was not until 1947 that stool testing, specifically FOBT, was used to detect CRC [34]. Also, the first colonoscopy was performed in 1969–over 50 years after the 1918 influenza pandemic [16,17]. A more contemporary public health crisis where we can say more definitively that CRC screening was affected is the 2013–2016 Ebola virus outbreak in Western Africa. As there is no published literature on this specific topic, and CRC is less common in Western Africa compared to other parts of the world, we can infer that any lockdown imposed during this time caused any scheduled CRC screenings to be delayed [35,36]. Ultimately, there truly is no other situation quite like the COVID-19 pandemic that affected CRC screening so drastically. The fact that there has never been a similar situation that affected CRC screening like COVID-19 has, is why it has been relatively difficult for healthcare providers to make decisions about how best to proceed in these times. One can expect that in the aftermath of the COVID-19 pandemic, healthcare in general and screening systems for CRC will improve to deal with future disasters like the current pandemic.

## 4. Resuming CRC Screening during COVID-19 and Beyond

Given the massive delays in screening with traditional methods, some healthcare centers looked toward allowing more CRC screenings to take place after initial waves of COVID-19. One study involving four healthcare locations in the UK was conducted to determine the safety of performing CTCs during the downturn period (May to July 2020) after the UK’s initial peak period of COVID-19. CTC is a faster procedure compared to colonoscopy and lends itself to social distancing, given that the process involves a computed tomography (CT) scan where no patient-staff physical contact is necessary. The study was done safely as no patients or staff involved in the CTCs were found to have COVID-19 after the test [37]. Additionally, CCE involves minimal physical contact between patients and staff; in fact, only one medical provider is needed to administer the test to a patient that can also be done remotely [38]. Note that throughout the pandemic, the use of telemedicine picked up to meet the needs of the healthcare system. This approach extends to CRC screening, where telehealth appointments regarding CRC screening have increased [39,40]. Telehealth visits are still ongoing, even as in-person CRC screenings in hospitals or other medical settings are starting to resume [41,42,43]. These appointments provide a platform for doctors to discuss available screening options with patients during these circumstances.

More importantly, the use of FIT is arguably the best alternative to, or prior to, colonoscopy procedure during the COVID-19 pandemic. FIT detects hidden blood in the stool, an early sign or warning of a silent cancerous lesion in the colon and/or rectum. To have maximum effectiveness, FIT must be done once a year. With the use of kits containing directions and materials, patients can simply do the test at home and then send it to their medical provider [15,44]. FIT can serve as a triage to colonoscopy or other screening tests. One study in the UK found that using FIT during the pandemic allowed for improved utilization of resources regarding colonoscopy; specifically, by using the FIT before colonoscopy, they found that the number of patients referred for a colonoscopy decreased by 28% while still maintaining the amount of CRC diagnosis [45]. This trend was found when they looked at the number of colonoscopies performed over the course of 26 March 2020 to 2 July 2020 compared to 1 October 2019 to 31 December 2019, where there was no statistically significant difference in the number of CRC diagnoses. By not having colonoscopies (when they are not strictly necessary), it saves time and money for providers and patients who would have had to undergo colonoscopy if they had not first received FIT [45]. To give some context, in the UK, it costs the National Health Service (NHS) approximately £372 for a colonoscopy, as compared to £5 for each FIT [46]. While in the U.S., a colonoscopy costs an average of $2750 for a patient, and depending on the type of insurance they have, they could be paying varying amounts; or, with no insurance, patients would have to pay the full amount [47]. In 2017, NHS funded a study known as NICE FIT study. It was conducted in 50 hospitals in UK with 9822 subjects to assess effectiveness and the accuracy of the home-based stool testing kits (FIT) to reduce invasive screening procedures like colonoscopies and results have recently published [46,48]. According to the results, the sensitivity of FIT for CRC in high-risk patients was 97.7% and 92.2% at cut-off values of 2 and 10 μg hemoglobin per g of stool sample. Similarly, it showed 94.3% and 86.6% sensitivity in low-risk patients at the same cut-off. These results confirm the effectiveness of FIT use in investigations for CRC screening.

Similarly, Issaka et al. used a developed simulation model to determine that during a period of fewer CRC screenings—i.e., the COVID-19 pandemic—an increase in FIT tests is associated with increased colorectal screenings and diagnoses. More specifically, they estimated that there would be an increase of about 2836 CRC diagnoses and 588,844 CRC screenings, totaling up to about 68.9% of CRC diagnoses being in the early stage of cancer [33]. If this period of delayed CRC was extended, it was estimated that the number of screenings and diagnoses would also go up [33]. Thus, while a FIT test does not provide all the benefits of a colonoscopy, it is a crucial tool in distanced CRC screening to close the gap during the COVID-19 pandemic. Not only is this type of test beneficial for maintaining the presence of screening, but it can also help to reduce racial and socioeconomic disparities in that it provides an at-home, lower-cost alternative to the traditional colonoscopy [33]. Although racial disparity is not the main focus of this review, it should be noted that this existing issue of disparity in cancer prevention in minority and ethnic populations, was clearly exposed during the COVID-19 pandemic. Carethers et al. have discussed the beneficial role of at-home tests during the pandemic in decreasing overall disparity in medically underserved populations [49]. These stool-based non-invasive tests should be further considered and implemented as part of regular CRC screening programs to prevent countless deaths. Beyond their immediate CRC benefit, they lead to changes done in the realm of public health and overall patient well-being.

While FIT can and likely should be used more given the COVID-19 pandemic to triage patients, it cannot replace colonoscopy procedure and its functional utility. Furthermore, challenges people face in receiving CRC screening pre-pandemic have been amplified even more during the pandemic. For instance, people who might have a harder time making themselves available to come get a colonoscopy, due to work obligations, for instance, are even more likely to not get a colonoscopy during the pandemic due to fear of contracting COVID-19. For example, one study found that nearly 20% of people in their CRC sample said that they would not be as likely to go do a screening test during the pandemic as compared to before the pandemic [50]. Also, the fact that stress levels in the general population have increased because of the pandemic could be another reason why people are less likely to come to a hospital/medical center for any kind of CRC screening [51]. Even beyond CRC, patients were still reluctant for any kind of healthcare appointment in general. This reluctance can be due to multiple factors mentioned before, such as stress, fear, or overall emotional unsettledness. In the same study, it was shown that a significant portion of the population, about 36.9%, have been reported to miss at least one healthcare appointment during the pandemic [51]. Therefore, there is even more uncertainty around the impact of these missed appointments even beyond CRC. This can be seen with overall delayed diagnosis and missed diagnosis in other types of cancers as well, which again have an increased death rate associated with prolonged, late diagnosis of the disease.

As stated, and implied previously, there is no published literature detailing the effects of the 1918 flu pandemic on colorectal cancer incidence in the years following the 1918 flu pandemic. One reason could be lack of advanced medical care and unavailability of screening methodology almost a century ago. Of course, somewhat of a parallel between the 1918 flu pandemic and COVID-19 is that people were afraid to interact with others (outside of their homes), including physicians, for fear of contracting disease at the beginning and peak of the pandemic. The fact that present-day medical technology itself collapsed during the initial stages of COVID-19 pandemic (meaning it could not be best-utilized given the limitations of the pandemic–social distancing, lockdowns, etc.) made it difficult to conduct routine screenings to obtain early cancer diagnoses.

Table 3 below summarizes the main findings of the studies related to COVID-19 included in this review.

## 5. Conclusions

There is no denying that the effects of COVID-19 on cancer screening are extreme. With interruptions and delays in screening tests, CRC mortality rates have been predicted to increase. Although the outlook of CRC seems ominous, one can take away countless lessons from the COVID-19 pandemic in terms of screening, diagnosis, and overall prevention of CRC. One key component to consider for the future of CRC screening and diagnosis is more utilization of alternative approaches, such as FIT tests. This remote option gives patients a lot of flexibility with their screening, as they are able to take the test safely in the comfort of their own home. It can also potentially provide a better allocation of resources, in terms of the fact that it can help prioritize patients who need colonoscopy over others due to the relative severity of their disease stage. Along with that, the accessibility of FIT tests can improve racial health disparities. Even as regular screening practices start to pick back up again as COVID-19 vaccines are being administered in many developed countries, the pandemic has still altered the framework through which healthcare providers view CRC screening. A lot has been learned and there are many key takeaways. One of these takeaways is that while colonoscopy will always be the gold standard in terms of CRC screening, FIT tests and other screening methods have significant strengths and unique attributes that make them utilizable and optimal in different situations.

Prediction models were utilized to have a sense of the extent to which delayed screenings could impact the population. In past pandemics, such as the 1918 flu pandemic, there were no clear data to demonstrate if the same trends occurred in terms of increased deaths due to missed screenings, and delayed diagnoses of CRC. The same could be said about the Ebola virus outbreak, a more contemporary epidemic, because there is no literature about these exact trends either. It is extremely important that we must use what we know about how COVID-19 affected CRC to prepare for and prevent human suffering in future pandemics and other public health crises. Doing so could help prevent the effects on CRC of a future event from being as catastrophic as early diagnosis, along with proper intervention, saves lives. More recently, as COVID-19 vaccines become more and more available, regular screening practices have started to pick back up again. However, the consequent delays previously mentioned still take a toll on human lives in terms of delayed diagnosis and advancement of CRC. One can hope that lessons learned from the current pandemic will make us efficient enough to respond by strategizing existing resources and rationalizing health systems to optimize cancer screening and cancer therapies by making smart decisions to protect human lives if we must go through this crisis again.

This manuscript provides a perspective for the medical community regarding the continuation of screening programs for chronic and fatal diseases (i.e., different cancers including CRC) even during the pandemic times that can lead to a decrease in mortality for these conditions that otherwise was increased due to diagnosis in the late stage of these diseases. There are some strengths and limitations to this review. The main point is that this manuscript compares CRC screening during the COVID-19 pandemic to CRC screening in the 1918 flu pandemic and the Ebola virus outbreak, which has never been done previously in other reviews. Also, this review compiles qualitative information pertaining to several countries, giving it a more global perspective. One of the main limitations to this review is that the data for CRC screening during and in the years following the 1918 flu pandemic is very limited (mainly due to the fact that screening was not prevalent at that time). Also, the COVID-19 pandemic is still ongoing, so many of the statistics, as well as model predictions, are limited to the point in time of the pandemic at which they were gathered and conducted.

The consequence of delayed and missed CRC diagnoses will, unfortunately, be felt for years to come by everyone in the world. There is still a lot left to learn and discover in terms of CRC screening, and the challenges that have had to be overcome in the present pandemic show that there certainly is more capacity to improve CRC screening. Unfortunately, there have been fatal implications due to COVID-19. However, by reflecting on and analyzing all of what COVID-19 affected in terms of CRC screening (and all other areas), the healthcare population/industry, researchers, etc., can form plans and solutions to help prevent these events from reoccurring.

## Figures and Tables

**Table 1:** Number of Cases and Deaths of 1918 Flu and COVID-19 Pandemics.

Pandemics	Total Cases		Total Deaths	
	World	USA	World	USA
**1918 Flu ^a^**	500,000,000	29,400,000	50,000,000	675,000
**COVID-19 ^b^**	259,819,859	48,107,102	5,180,137	775,628

**^a^** Source: https://www.cdc.gov/flu/pandemic-resources/1918-pandemic-h1n1.html (accessed on 21 November 2021). **^b^** Source: https://coronavirus.jhu.edu/map.html. (As of 25 November 2021).

**Table 2:** Most Common Types of Colorectal Cancer (CRC) Screening Tests.

Type of Screening Method	Invasiveness	Method	Location to Conduct (Hospital or Home)	Frequency
Colonoscopy	Invasive	Colonoscope is used to examine colon and rectum; image transmitted	Hospital (needed an expert GI physician)	Once every 10 years (if normal)
Flexible Sigmoidoscopy	Invasive	Same procedure as a colonoscopy except that only last one third of the colon is examined (instead of both the colon and rectum)	Hospital (needed an expert GI physician)	Once every 5 years
Colon Capsule Endoscopy	Slightly invasive	Unlike colonoscopy, orally ingested pill camera is used to transmit images	Home (needed GI physician to evaluate images)	Not established yet
Guaiac-Based Fecal Occult Blood Test	Non-invasive	Stool test; detects blood in stool using the chemical guaiac	Home	Once every year
Fecal Immunochemical Test (FIT)	Non-invasive	Stool test; detects blood in stool using antibodies	Home	Once every year
FIT-DNA Test	Non-invasive	Stool test; detects altered DNA	Home	Once every 1 or 3 years
CT Colonography	Non-invasive	Technology like X-rays used to capture complete images of the colon	Hospital (needed expert to read)	Once every 5 years

**Table 3:** Summary of Main Findings of Studies Relevant to Colorectal Cancer Screening and COVID-19 Pandemic.

Authors & Reference#	Journal	Year Published	Main Findings
Johnson BA et al. [6]	Am J Surg	2020	A delay in surgeries for breast, lung, and colon cancer during the COVID-19 pandemic could decrease the chance of survival for patients.
Lantinga MA et al. [26]	Endoscopy	2021	Data from a database, encompassing 15 hospitals in the Netherlands were analyzed. Comparing the 15 March 2019–25 June 2019 period to the 15 March 2020–25 June 2020 period, it was seen that gastroscopies decreased by 57% and colonoscopies decreased by 55%.
Wassie MM et al. [27]	JGH Open	2021	A retrospective data analysis was performed to compare colonoscopy data from April–June 2019 to April–June 2020 in South Australia. Colonoscopies decreased by 51.1% in the COVID-19 time period, and 46.1% of colonoscopies were delayed by at least 6 months.
London JW et al. [28]	JCO Clin Cancer Inform	2020	Data from the TriNetX data network, encompassing 20 medical centers and more than 28 million patients total, were analyzed. Comparing March 2019 to March 2020, there was a 38.4% decrease in CRC screenings. Comparing April 2019 to April 2020, there was an 84.5% decrease.
Domper Arnal MJ [29]	Presented at: UEG Week; 3–5 October 2021 (virtual meeting)	2021	An unpublished study of hospitals in Spain found there was a 40.4% decrease in CRC diagnoses in March 2020–February 2021, as compared to March 2019–March 2020.
Maringe C et al. [30]	Lancet Oncol	2021	This UK population-based modelling study aimed to estimate the effects of COVID-19-related delays of CRC screening. Data for 24,975 people with CRC was collected, and the estimation of additional death toll was 1445 to 1563 people up to five years post-diagnosis.
Yong JH et al. [31]	J Med Screen	2021	This study utilized microsimulation models for two types of cancers, including CRC, to determine what effects COVID-19 could have on CRC in Canada. With a 6-month delay in regular screening, CRC incidence and death would increase by 2200 people and 960 people, respectively.
Kregting LM et al. [32]	Br J Cancer	2021	Microsimulation models were used to simulate screening restart strategies for different types of cancers. If delays (due to the pandemic) were not caught up with, the CRC death rate would increase by 2.5 per 100,000 people in the next 10 years.
Issaka RB et al. [33]	JAMA Netw Open	2021	A simulation model was used to predict CRC outcomes between 2020 and 2023 in the U.S., including delays associated with the COVID-19 pandemic. The model showed that there would be 1,176,942 to 2,014,164 less CRC screenings and 8346 to 12,894 fewer CRC diagnoses. Also, an increase in FIT could cause increases in CRC screenings (655,825) and diagnoses (2715).
Peprah D et al. [37]	Br J Radiol	2021	Evaluation of CTCs conducted from May to July 2020 at four English hospital trusts was performed. 224 patients were scanned; of these, 169 who were followed up with on the phone reported no COVID-19 symptoms within 14 days of the test.
MacLeod C et al. [38]	Colorectal Dis	2020	Colon capsule endoscopy is a screening method that can be carried out safely during the COVID-19 pandemic and can be used to triage for colonoscopy.
Wahezi SE et al. [39]	Best Pract Res Clin Anaesthesiol	2021	Telemedicine has greatly increased due to the COVID-19 pandemic and will continue to be practiced post-pandemic. More research on telemedicine is needed to better compare it to in-person visits.
Kadakuntla A et al. [40]	World J Gastrointest Oncol	2021	The delays in CRC screening during COVID-19 can be overcome by doing more stool-based tests, adjusting screening protocols, and implementing more telehealth. Telehealth has advantages including convenience and improving patient compliance.
Maclean W et al. [45]	Colorectal Dis	2020	An observational cohort study in the UK was conducted to evaluate how FIT could affect utilization of resources and triage to colonoscopy. They found using FIT decreased further progression to colonoscopy from 62% (pre-pandemic) to 34%, while no significant change in the diagnosis rate.
D’Souza N et al. [48]	Br J Surg	2021	From October 2017 to December 2019, a study of 50 hospitals in England including 9822 subjects showed that the sensitivity of fecal immunochemical tests for CRC was 97.7% and 92.2% at cut-off values of 2 and 10 μg hemoglobin per g of stool sample.
Carethers JM et al. [49]	Cancer Prev Res (Phila)	2020	Minority populations in the United States face additional challenges in receiving CRC screening (e.g., finances, transportation, etc.). Thus, the delays in CRC screening during the pandemic will increase their CRC risk.
Wilson R et al. [50]	Prev Med	2021	A cross-sectional online survey was conducted in the UK of over 7543 adults between August and September 2020, and follow-up interviews were conducted for 30 people. About 20% of participants indicated they would be less likely to go to a CRC screening test during the pandemic.
Mason MC et al. [51]	Cureus	2021	A cross-sectional study, done from January–April 2021, was performed (totaling 103 participants). Over 30% participants missed a routine colonoscopy during the pandemic.

## Data Availability

The data used in this paper come from sources found via searches on PubMed or Google (Google Scholar). See the References section for each source used.
